# Testosterone suppresses uropathogenic *Escherichia coli *invasion and colonization within prostate cells and inhibits inflammatory responses through JAK/STAT-1 signaling pathway

**DOI:** 10.1371/journal.pone.0180244

**Published:** 2017-06-30

**Authors:** Chen-Hsun Ho, Chia-Kwung Fan, Hong-Jeng Yu, Chia-Chang Wu, Kuan-Chou Chen, Shih-Ping Liu, Po-Ching Cheng

**Affiliations:** 1 Department of Urology, School of Medicine, Taipei Medical University, Taipei, Taiwan; 2 Department of Urology, Taipei Medical University-Shuang Ho Hospital, Taipei, Taiwan; 3 Department of Molecular Parasitology and Tropical Diseases, School of Medicine, Taipei Medical University, Taipei, Taiwan; 4 Center for International Tropical Medicine, School of Medicine, Taipei Medical University, Taipei, Taiwan; 5 Department of Urology, National Taiwan University Hospital and College of Medicine, Taipei, Taiwan; Northwestern University, UNITED STATES

## Abstract

Prostatitis is a common condition in adult men of all ages. Uropathogenic *Escherichia coli* (UPEC) are most frequent pathogen involved in bacterial prostatitis by refluxing the infected urine into prostatic ducts and resulting in an ascending urethral infection. However, the study about the mechanisms of UPEC to invade, replicate and persist in normal prostate epithelial cell is only few. Given the fact that UPEC is pathogen most frequently involved in prostatitis and that testosterone has been demonstrated to attenuate prostate inflammation caused by other etiologies. In this study we investigated whether the testosterone reduces the prostatitis and related mechanism by regulating IFN-γ/STAT1 signaling pathway. In the current study aimed to clarify whether testosterone influences the process of UPEC-induced prostate inflammation and invasion into the prostate epithelial cells. In addition, we set up a normal prostate cell model for UPEC infection to evaluate the ability to invade the urothelial cells as well as the colonization of intercellular bacterial communities in vitro. By using the model, we examine the effects of testosterone to suppress effectively the invasion and survival of UPEC in the prostate cells, and inhibit LPS-induced inflammatory responses through the JAK/STAT1 pathway have also been indicated. Our results demonstrated testosterone not only suppressed the invasion and colonization of UPEC, but also inhibited the expression of pro-inflammatory IL-1β, IL-6 and IL-8 cytokines expression induced by UPEC in a dose-dependent manner. We found the effective dose of testosterone to suppress UPEC infect prostate cells may be appropriate under 40μg/ml. Our data also revealed 20μg/ml testosterone treated PZ-HPV-7 cells significantly suppressed the LPS-induced JAK/STAT1 pathway and inflammatory responses, and reached to maximal effects at 40μg/ml treatment. These results indicate that testosterone plays an anti-inflammatory role in LPS-induced prostate cell inflammation by down-regulating JAK/STAT1 signaling pathway. Interestingly, the JAK inhibitor and testosterone for 24hr pretreatment rather markedly induced the colonization of UPEC in the PZ-HPV-7 cells. Based on the above data, the suppression of UPEC colonization in the prostate cells by testosterone seems to be unrelated with JAK/STAT signaling pathway, whereas the JAK may involve into the UPEC infection. Summing up these data, our findings have demonstrated the suppressive effects of testosterone on the invasion and survival of UPEC and induced inflammation in prostate epithelial cells. These findings indicate the action mechanism of testosterone as an anti-inflammatory mediator in the prostate cells is regulated through JAK/STAT1 signaling pathway, may be beneficial in treating prostate inflammation. Altogether, this study has provided the possibility that using testosterone in the prevention and clinical treatment of prostatitis is a new direction.

## Introduction

Uropathogenic Escherichia coli (UPEC) is the most common causative microorganism of urinary tract infection and accounts for the majority of acute and chronic bacterial prostatitis. With the expression of multiple virulence factors, such as fimbriae, lipopolysaccharide (LPS), and toxins, UPEC can trigger a series of host inflammatory responses, including cytokine production, neutrophil influx, and the exfoliation of uroepithelial cells [1]. Moreover, a substantial body of evidence has demonstrated that UPEC is capable of invading the epithelial cells of urinary bladder and forming a biofilm-like intracellular bacterial community [2]. This mechanism enables UPEC to evade host defense and antibiotic therapy and serves as a reservoir for recurrent or chronic infection. The intracellular colonization of UPEC has also been also demonstrated in prostate epithelial cells [3]. A recent study demonstrated the ability of as many as 58 UPEC strains to adhere to and invade normal human prostate cells with high efficiency [4]. These strains are capable of activating mitogen-activated protein kinase and NF-κB signaling pathways in the prostate RWPE-1 cell line, inducing release of proinflammatory cytokines, IL-6 and IL-8. [4]. An animal study demonstrated a high prevalence of UPEC persistence in the prostate tissue after 14 day of bacterial infection, suggesting UPEC colonization could be a frequent sequela of acute prostatitis [5].

It is well known that testosterone deficiency is associated with increased systemic inflammation and predisposes to many disorders related to inflammation [6, 7]. It was shown that low testosterone increases the risk of male accessory gland infection, including prostatovesiculitis and prostate-vesiculo-epididymitis [8], and transdermal testosterone supplement has a favorable effect on sperm quality in these men with male accessory gland infection and concomitant hypogonadism [9]. A more recent study also demonstrated that men with testosterone deficiency have a higher prevalence of chronic prostatitis/chronic pelvic pain syndrome [10]. On the other hand, animal studies demonstrated that high fat diet-induced testosterone deficiency leads to prostate inflammation, which can be reversed by testosterone supplementation [11, 12].

The Janus kinase (JAK) and signal transducer and activators of transcription (STAT) pathway is critical for the signaling of the modulatory mechanism by which cytokines contribute to the progression of inflammatory diseases [13]. Previous studies have proved that through suppressing the activation of IFN-γ/JAK/STATs pathway, LPS-induced inflammation could be effectively reduced via attenuating the Reactive oxygen species (ROS) and cytokines production. [14–16]. Many pathogenic infections can induce inflammation by activating the JAK / STAT pathway [17], and even more, they by mediating these pathways to evade the immune responses. Recent reports showed H. pylori may via negative regulator of IFN-γ/STAT1 signaling to developing resistance to escape from IFN-g–mediated bacterial clearance [18]. Another report also showed Newcastle disease virus V protein can target phospho-STAT1 degradation to block IFN-α signaling [19]. It has been indicated the levels of IFN-γ, TNF-α and IL-1β were elevated in the semen of CP / CPPS-suffering patients [20]. IFN-γ/STAT1 signaling pathway has also been shown plays an important role in the immune responses to prostatitis in experimental autoimmune prostatitis mouse model [21]. However the researches about whether the testosterone reduces the prostatitis and the regulated mechanism relate to IFN-γ/STAT1 signaling pathway both remain lacked.

Given the fact that UPEC is pathogen most frequently involved in prostatitis and that testosterone has been demonstrated to attenuate prostate inflammation caused by other etiologies, the current study aimed to clarify whether testosterone influences the process of UPEC-induced prostate inflammation and the invasion into the prostate epithelial cells. In addition, we built a specific GFP-UPEC-infected prostate cell model to provide a platform for evaluating the effects of testosterone or other medicine on prostatitis treatment in the future.

## Materials and methods

### Cell culture

PZ-HPV-7 cell line is a normal human prostate epithelial cell line acquired from the American Type Tissue Culture Collection (ATCC, Rockville, MA, USA). Cells were maintained in Keratinocyte-serum-free medium (K-SFM) supplemented with bovine pituitary extract (50 μg/ml) and epidermal growth factor (5 ng/ml) (Gibco/Invitrogen, Grand Island, NY, USA) at 37°C in a humidified atmosphere of 5% CO_2_. The medium was replaced every 2 or 3 days.

### Bacterial strains

As the model, we used uropathogenic E. coli (UPEC) CFT073 from ATCC 700928 [22]. UPEC was expressed green fluorescent by transformation with the pGFP plasmid (Clontech, Palo Alto, CA). Bacterial strains were cultured in LB broth supplemented with ampicillin (100 μg/ml) and incubated at 37°C overnight. Bacterial growth was determined spectrophotometrically at an optical density of 600 nm (OD_600_). Green fluorescent of pGFP was induced by addition of 5μM isopropyl-β-d-thiogalactoside (IPTG) to the growth medium [23]. For *in vitro* infections, bacteria were suspended in culture medium at multiplicity of infection (MOI).

### Drug treatments

In the UPEC infection model, PZ-HPV-7 cells were seeded into 12-well dishes (5 x 10^6^ cells/mL) and pretreated the next day with 20, 40, 60 μg/mL testosterone (5α-androstan-3β-ol-17β-one; Sigma-Aldrich, St. Louis, MO, USA) for 24 h prior to the bacterial infection. Drugs did not affect the viability or growth of *E*. *coli*. Each condition was measured in duplicate, and each experiment was performed at least thrice. For studying LPS-induced inflammation, cells were plated at a density of 1 × 10^6^ cells per well and treated with 5 μg/mL LPS (from *E*. *coli* 0111:B4; Sigma-Aldrich, St. Louis, MO, USA; protein content, < 1%) after co-incubating in the presence or absence of various concentrations (10, 20, 40 μg/mL) of testosterone for 24 h. In addition, another group of control cells were co-cultured in the presence of the same concentrations (10, 20, 40 μg/mL) of testosterone alone for 24 h for observing the effects of testosterone on JAK/STAT1 pathway. Signaling block experiments were performed by pre-incubation with 50 mM JAK inhibitor (Ruxolitinib; MedChem Express, Princeton, NJ, USA) or 50 mM STAT1 inhibitor (Fludarabine; Selleck Chemicals Inc., Houston, TX, USA) and testosterone for 24 h before the UPEC treatment [24, 25]. We also treated the cells with 50 mM JAK inhibitor or 50 mM STAT1 inhibitor alone for 24 h before UPEC treatment for observing the effects of UPEC infection.

### Bacterial infections and quantifying invasion and intracellular colonization

PZ-HPV-7 cells after testosterone treatment were infected with bacteria (MOI of 100) and incubated for 3 hr at 37°C with 5% CO_2_. After 3 hr infection, infected monolayers were washed 4 times with phosphate-buffered saline (PBS) and incubated for 30 min in growth medium containing gentamicin (100μg/ml; Sigma-Aldrich, St. Louis, MO). To measure bacterial invasion, cells were lysed and harvested by 0.5% trypsin (Gibco)–0.1% Triton X-100 (Amresco, Solon, OH, USA) than plated onto LB agar. Colonies were counted to quantify bound bacteria [26–28]. In colonization assay, UPEC-infected cells were be incubated 24hr after invasion, followed by washing, lysing, bacterial plating as described previously.

### Fluorescence microscopy for UPEC detection

To detect green fluorescent of UPEC, PZ-HPV-7 cells seeded onto 18mm coverslips were infected with pGFP-UPEC(MOI of 100) as previous described [26]. Coverslip were observed by fluorescence microscopy. To quantify invasion and colonization, images of 50 random fields of each coverslip were acquired.

### Flow cytometry

Cells after colonization assay were harvested by 0.25% trypsin-EDTA (Gibco/ Invitrogen, Grand Island, NY, USA) which was neutralized with 0.1% soybean trypsin inhibitor (Gibco/Invitrogen, Grand Island, NY, USA). These cells were transferred in eppendorf tubes and centrifugated at 1000xg for 10 min at 4°C. Samples were washed and resuspended in 0.22 μm filtered PBS. We monitored intracellular pGFP-UPEC at 488nm using BD-FACS VERSE (BD Biosciences, CA, USA).

### RNA isolation and RT-PCR

Total RNA was isolated from PZ-HPV-7 cells using TRIzol™ reagent (Life Technologies, Carlsbad, CA, USA). After treatment, cells were scraped with 1000μl of TRIzol™ reagent then mixed with 200μl chloroform. After centrifugation at 13,000xg for 15 min, the aqueous phase was collected and mixed with 500μl isopropanol. The supernatant was removed and the RNA precipitate dissolved in deionized water for quantification. Then, total RNA was converted to cDNA using a High-Capacity cDNA Reverse Transcription Kit (Applied Biosystems, Foster City, CA).Each cDNA pool was stored at -20°C until further real-time PCR. Real-time PCR, performed with 2X SensiFAST™ SYBR^®^ No-ROX Kit (Bioline, UK), was analyzed using the Roche LightCycler® 480 instrument (Roche Applied Science, Indianapolis, Ind.). qPCR reactions were carried out in a total reaction volume of 20 μl, containing 2x SensiFAST™ SYBR^®^ No-ROX Mix, 8 μM each of forward and reverse primers and 5 μl template cDNA. The primers used are: janus kinase 1(JAK 1), forward:5’-AGACTTGTGAATAC GTTAAAAGAAGGA-3’,reverse:5’-AAAGCTTGTCCGATTGGA TG-3’; janus kinase 2(JAK 2),forward: 5’-GATGGATGCCCAGATGAGAT-3’, reverse:5’-TTGATCCACTCGAAGAGCTAGA-3’; signal transducer and activator of transcription 1 (STAT1), forward:5’- GGAACTTGATGGCCCTAAAGGA-3’,reverse:5’- ACAGAGCCCACTATCCGAGACA-3’; Interferon gamma (IFN-γ), forward: 5’-CTTCCTCATGGCTGTTTCTG-3’, reverse:5’-TGTCACCATCC TTT GCCAG- 3’;interleukin-6 (IL-6), forward: 5'-AACAAGAAAGACAAAGCCAGAGT CC-3', reverse: 5'-TGATTTCAAGATGAATTGGATGGTC-3'; interleukin-1beta(IL-1β), forward:5'-GCTG AAAGCTCTCCACCTCAA-3',reverse:5'-GTATTGCTTGGGATCCACACTCT- 3';interleukin-8(IL-8), IL-8, forward: 5ꞌ-CACCTCAAGAACATCCAGAGCT-3ꞌ, reverse: 5ꞌ-CAAGCAGAACTGAACTACCATCG -3ꞌ,.18s were used as endogenous reference genes. 18s, forward: 5'-ACAATACAGGACTCTTTC GAG -3', reverse: 5'-AGCTTTTTAACTGCAGCAAC -3'. The real time PCR condition was 3 min at 95°C; 45 cycles for 10 seconds at 95°C, 30 seconds at 57°C. At the end of the program, a melt curve analysis was done. The relative expression of mRNA was calculated by using 2–ΔΔ threshold cycle (Ct) (Livak) method [29]. The RT-PCRs were performed in triplicate for each of the three independent samples.

### Cytometric bead array immunoassay

Medium of cells was centrifuged (13,000xg, 20 min, 4°C), and the supernatant was assessed using human inflammatory cytokines cytometric bead array (CBA, BD Biosciences, San Diego, USA) for the cytokines IL-8, IL-1β and IL-6. The cytokine capture bead, phycoerythrin (PE) detection reagent and recombinant standards or test samples were incubated together for 3hr at room temperature. Using FACSCanto flow cytometer (BD Biosciences, San Diego, USA) to acquire the data and analyzing by BD CBA Analysis Software to receive the graph [30, 31].

### Western blotting analysis

After treatment, cells were washed and rinsed with cold phosphate buffered saline (PBS). Proteins were harvested in RIPA lysis buffer (0.5 M Tris-HCl, pH 7.4, 1.5 M NaCl, 2.5% deoxycholic acid, 10% NP-40, 10 mM EDTA). Cell suspensions were centrifuged at 10,000 x g for 20 min at 4°C. The Bradford protein assay kit was used (BioRad, Hercules, CA, USA) to estimate the total protein content. Equal amounts of protein samples were electrophoresed in 12% sodium dodecyl sulfate-polyacrylamide gels and transferred to polyvinylidene difluoride membranes (Millipore, Billerica, MA, USA). Nonspecific binding sites on the membranes were blocked using 15 mM Tris/150 mM NaCl buffer (pH 7.4) containing 5% nonfat milk overnight at 4°C. Membranes were probed with 1:1000 dilutions each of anti-JAK1 (R & D Systems), anti-JAK2, anti-STAT1 (Cell Signaling, Beverly, MA), anti-IL-6 (Cell Signaling), anti-IL-1β, and anti-β-actin (Cell Signaling) antibodies at room temperature for 1 h. After incubation with the appropriate secondary horseradish peroxidase-conjugated IgG antibody (R & D Systems) for 30 min at room temperature, the protein bands on the membrane were detected using ECL-Plus western blot detection system (GE Healthcare UK Ltd., UK) according to the manufacturer’s instructions. All experiments were replicated at least thrice. The results of typical experiments are shown. Graphical analysis of band density was performed using the ImageJ software (version 1.41o) (National Institutes of Health, Bethesda, MD) (http://rsb.info.nih.gov/ij/).

### Statistics

Data were expressed as mean ± standard deviation (SD). Analysis of variance was used to analyze the differences in surface marker expression between various treatment groups and controls. Statistical differences between groups were determined using Student’s t test. A p value of, <0.05 was considered significantly.

## Results

### Testosterone suppresses the invasion and colonization of UPEC

**Fig 1:**
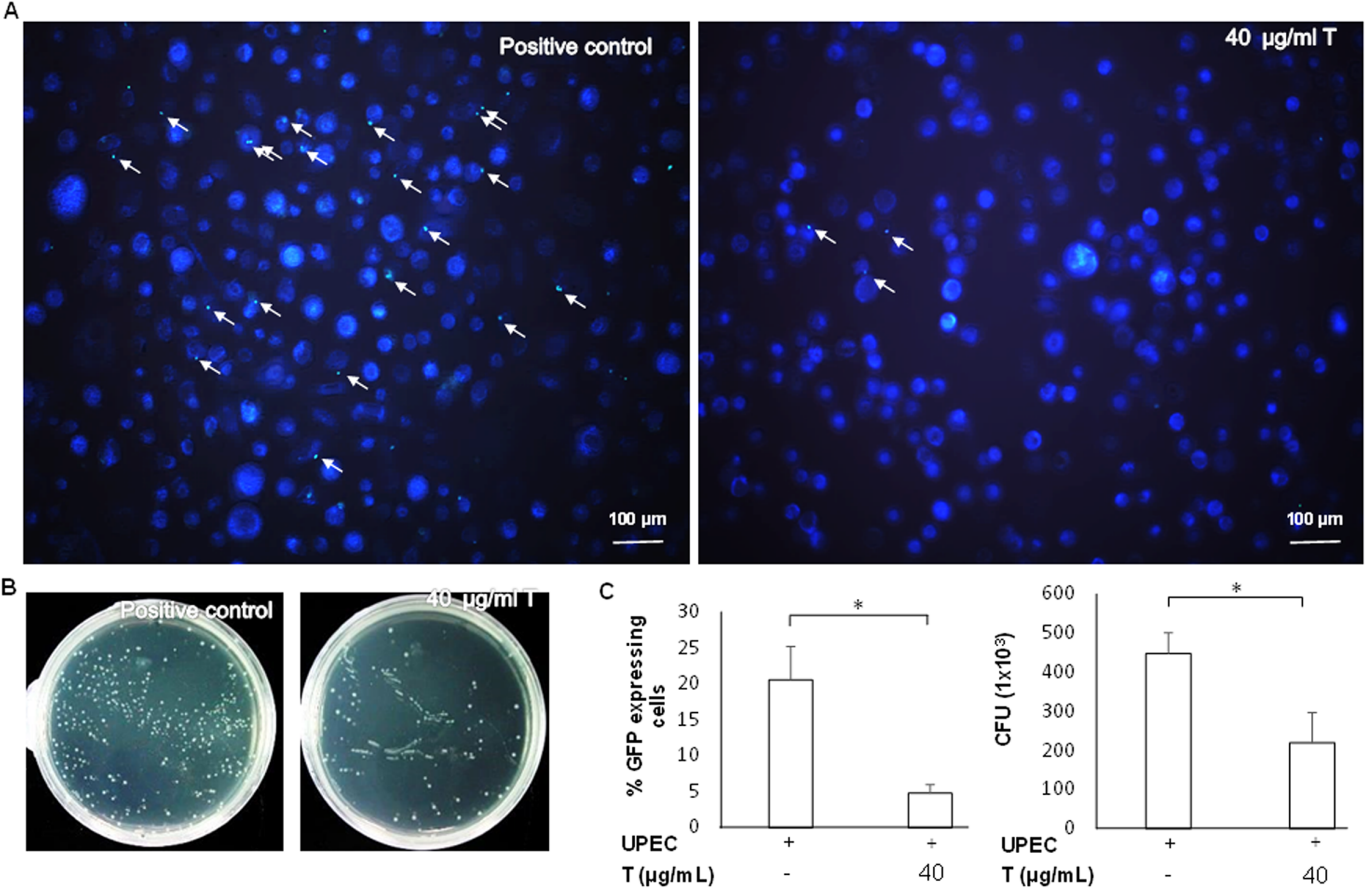
Effect of testosterone on UPEC invasion in PZ-HPV-7 cells. PZ-HPV-7 cells were pretreated with testosterone (T) for 24 h, and then infected with pGFP-UPEC (MOI; 100) as described in the Materials and methods. pGFP-UPEC were observed using fluorescence microscopy 3 h post-infection of prostate cells (objective 100x, scale bars 100 μm) (A), plated on LB agar (B), and the numbers of infected cells in (A) and (B) were quantified using Image J software (C, D). Sites of UPEC invasion in cells are shown with arrows. Colony forming units (CFU) were acquired after plating out ten-fold dilutions of infected cells after lysis. Data are expressed as the mean ± SD of three independent experiments. * p < 0.05, compared to the respective positive control groups.

**Fig 2:**
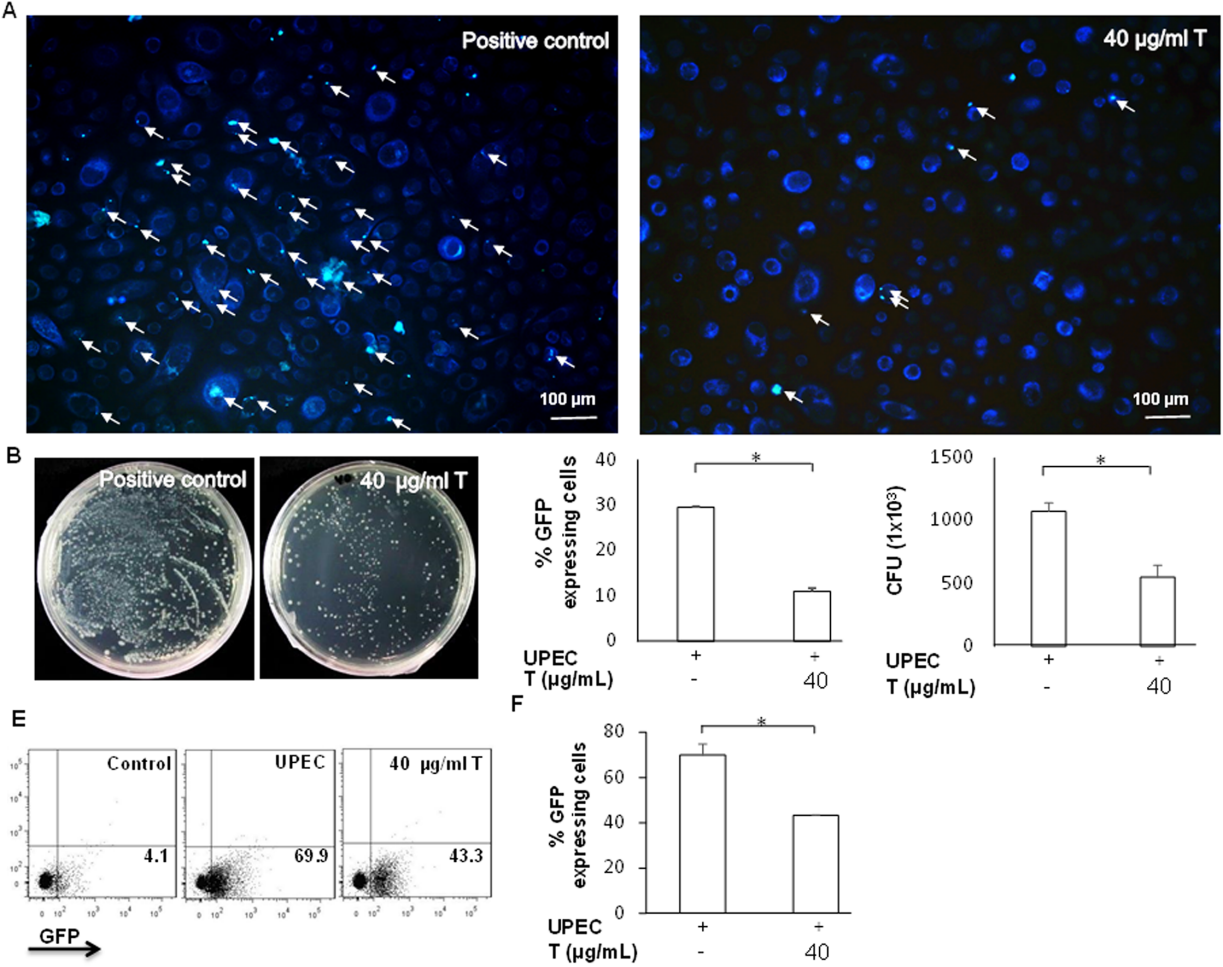
Effect of testosterone on UPEC colonization in PZ HPV-7 cells. PZ-HPV-7 cells were pretreated with testosterone (T) for 24 h and then infected with pGFP-UPEC (MOI; 100) as described in the Materials and methods. pGFP-UPEC colonization was observed using fluorescence microscopy (objective 100x, scale bars 100 μm) (A), plated on LB agar (B), and the measured values (C, D) within prostate cells of (A) and (B) were detected 24 h post-infection. Sites of UPEC invasion in cells are shown with arrows. Flow cytometry (E, F) was also used to analyze the cell ratios of UPEC colonization. Data from three separate experiments are shown as dot-blots for all analyzed samples, and one representative per experiment is shown. Numbers in the right lower quadrant show the frequency of cells expressing GFP in the quadrant of total cells. Colony forming units (CFU) were acquired after plating ten-fold dilutions of the infected cells post-lysis. Data are expressed as the mean ± SD of three independent experiments. * p < 0.05 compared to the positive control group.

Green fluorescent UPEC were observed by fluorescence microscopy, which confirmed that UPEC-infected cells are useful for examining UPEC invasion and colonization of prostate cells. We examined the role of testosterone in invasion and colonization of UPEC. The expression of pGFP-UPEC decreased significantly when cells were pretreated with 40 μg/mL testosterone for 24 h (p < 0.05 compared to the positive control) (Fig 1A–1D). Consistent with these results, testosterone was also able to significantly reduce UPEC colonization within prostate cells 24 h post-infection (p < 0.05 compared to the positive control) (Fig 2A–2D). Flow cytometry was used to precisely quantify UPEC colonization of prostate cells. Results showed that pretreatment with testosterone strongly suppressed UPEC colonization in prostate cells (p < 0.05) (Fig 2E and 2F). In summary, these results demonstrated that testosterone is able to effectively reduce the invasive and colonization abilities of UPEC in the prostate cells.

### Testosterone suppresses the colonization and inflammatory cytokine expressions induced by UPEC in dose-dependent manner

**Fig 3:**
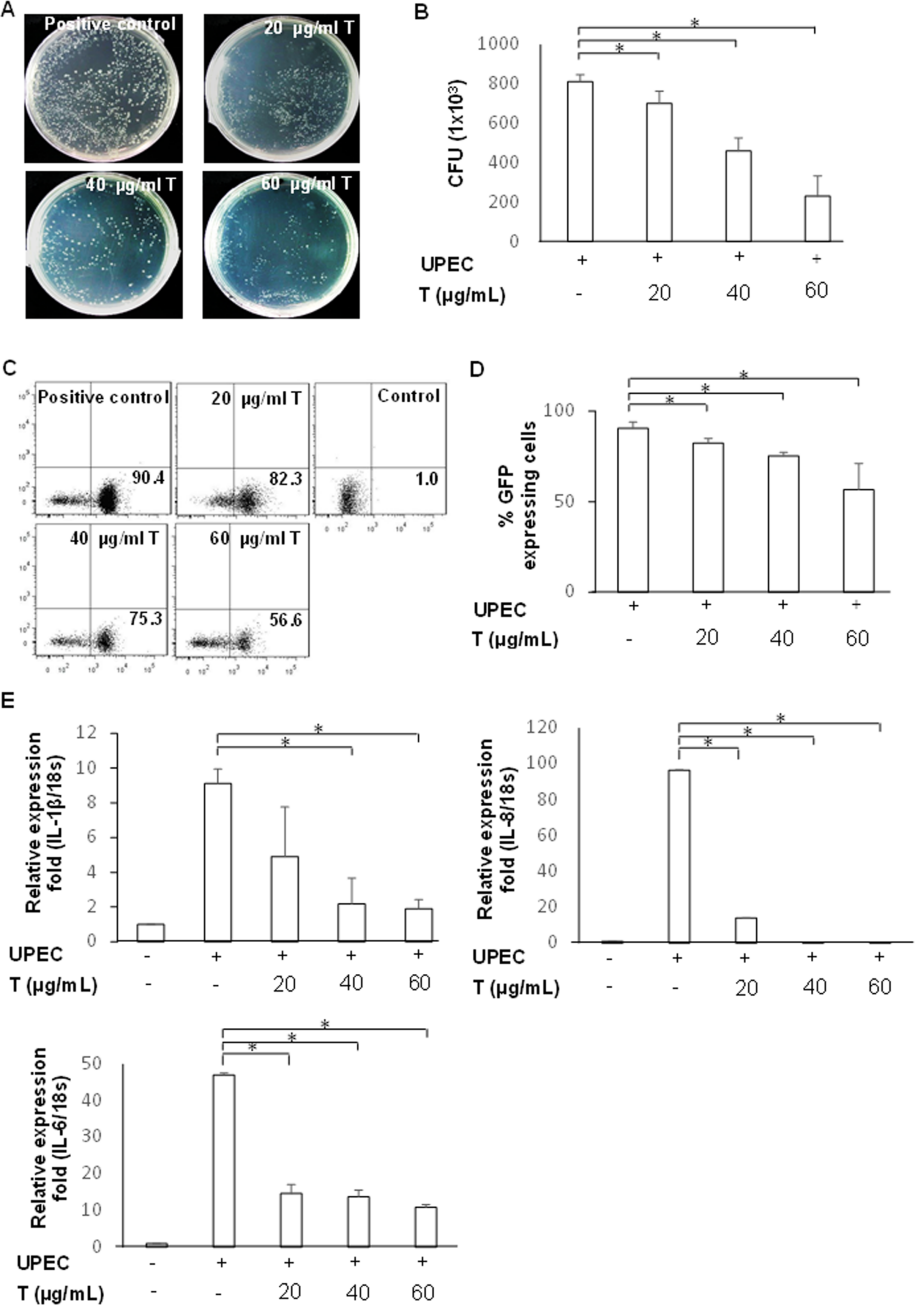
Effect of testosterone pretreatment on UPEC colonization and inflammation induction in prostate cells. PZ-HPV-7 cells were pretreated different doses of testosterone (T) (20, 40, and 60 μg/mL) for 24 h and then infected with UPEC as described above. Twenty-four hours post-infection, all groups of infected cells were lysed and plated on LB agar (A, B) or detected by flow cytometry (C, D) to analyze the cell ratios of UPEC colonization. Colony forming units (CFU) were acquired after plating ten-fold dilutions of infected cells post-lysis. Data from three separate experiments are shown as dot-blots for all analyzed samples, and one representative per experiment is shown. Numbers in the right lower quadrant show the frequency of GFP-expressing cells in the quadrant of total cells. The mRNA levels of *IL-6*, *IL1B*, and *IL-8* cytokines were measured using quantitative polymerase chain reaction (qPCR) in cells treated with different doses of T (E). Data from 3 separate experiments are expressed as the mean ± SD. * p < 0.05, compared to the respective positive controls.

To determine the effective doses for reducing UPEC colonization, cells were incubated with 20, 40, and 60 μg/mL testosterone prior to the infection. Pre-addition of 20–60 μg/mL testosterone suppressed UPEC colonization compared to that of the positive control (p < 0.05) (Fig 3A–3D). However, treatment with 60 μg/mL testosterone affected the physiology of the prostate cells and drastically reduced the cell number. Therefore, the effective dose of testosterone that suppresses UPEC infection in prostate cells was ≤ 40 μg/mL. The induction of inflammation significantly increased the expression of pro-inflammatory cytokines *IL-6*, *IL1B*, and *IL-8* from the prostate cells. To determine whether testosterone can also affect the inflammation induced by UPEC infection, PZ-HPV-7 cells were infected with UPEC as described above after dose-dependent testosterone treatment. Twenty-four hours post-infection, the mRNA levels of *IL-6*, *IL1B*, and *IL-8* were lower in the testosterone-pretreated cells in a dose-dependent manner (p < 0.05) compared to those of the positive control (Fig 3E). These results showed that testosterone treatment reduced both UPEC colonization and the post-infection inflammatory response.

### Testosterone decreases LPS-induced exogenous inflammatory cytokine secretion

**Fig 4:**
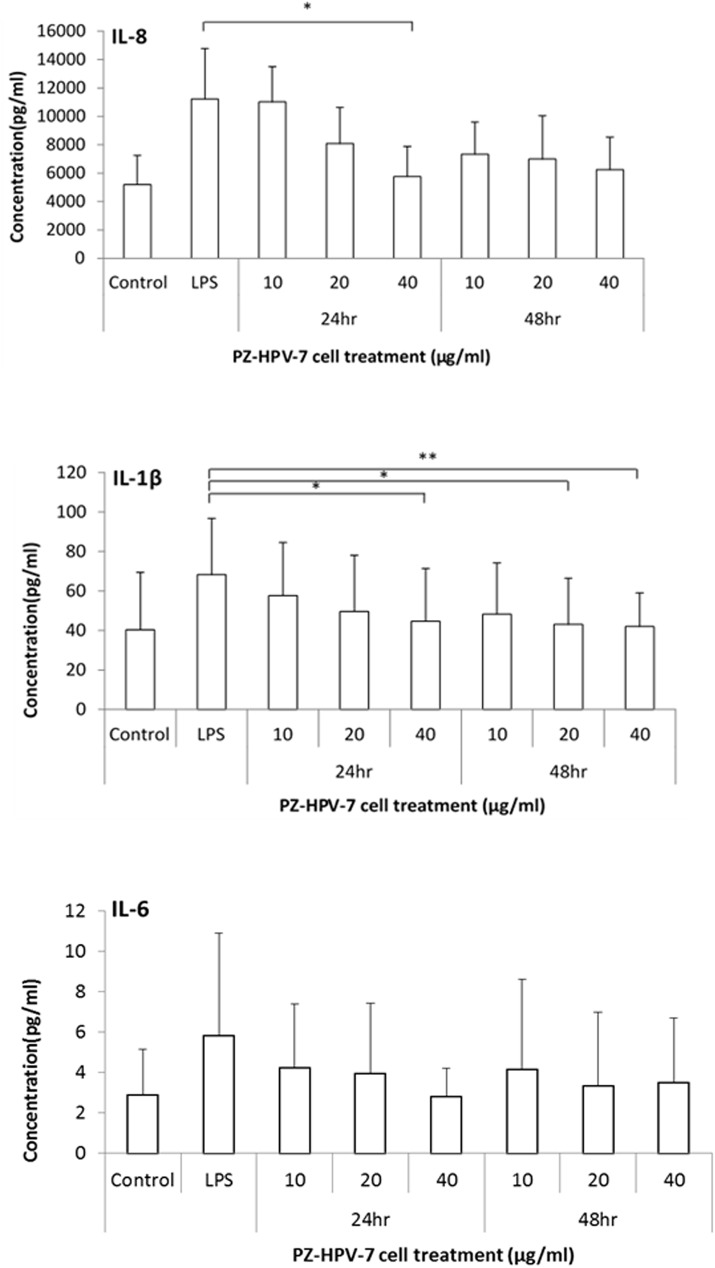
Testosterone affected the secretion of inflammatory cytokines in the LPS-stimulated prostate cells. After treating with different doses of testosterone (T) (10, 20, and 40 μg/mL) for 24 h and 48 h, the levels of cytokines IL-8, IL-1β, and IL-6 released from LPS-stimulated PZ-HPV-7 cells were measured using CBA. Cells with LPS alone stimulation (5 μg/mL) were used as the positive control. Data are expressed as the mean ± SD from 3 separate experiments. * p < 0.05, ** p < 0.01, compared to the positive control.

Next, we attempted to understand the role of testosterone in the induction of inflammatory response in prostate cells. For this purpose, we investigated whether testosterone treatment could reduce the exogenous secretion of LPS-induced inflammatory cytokines in prostate cells. CBA analysis of the supernatants obtained from cells cultured with different doses of testosterone confirmed that testosterone inhibited LPS-stimulated inflammation (Fig 4). After treatment with 40 μg/mL testosterone for 24 h, there was a significant decrease in the levels of LPS-stimulated secretion of extracellular IL-8 (p < 0.05). The levels of extracellular IL-1β were markedly reduced upon treatment with 40 μg/mL testosterone for 24 h and with 20 and 40 μg/mL testosterone for 48 h (p < 0.05, 0.05, and 0.01, compared to LPS alone treatment). Secretion of both cytokines was significantly reduced in a dose- and time-dependent manner after testosterone treatment, whereas there was no statistical difference in IL-6 secretion in all the treated groups compared to the positive control.

### Testosterone reduces inflammatory cytokine expression by downregulating the JAK/STAT1 signaling pathway

**Fig 5:**
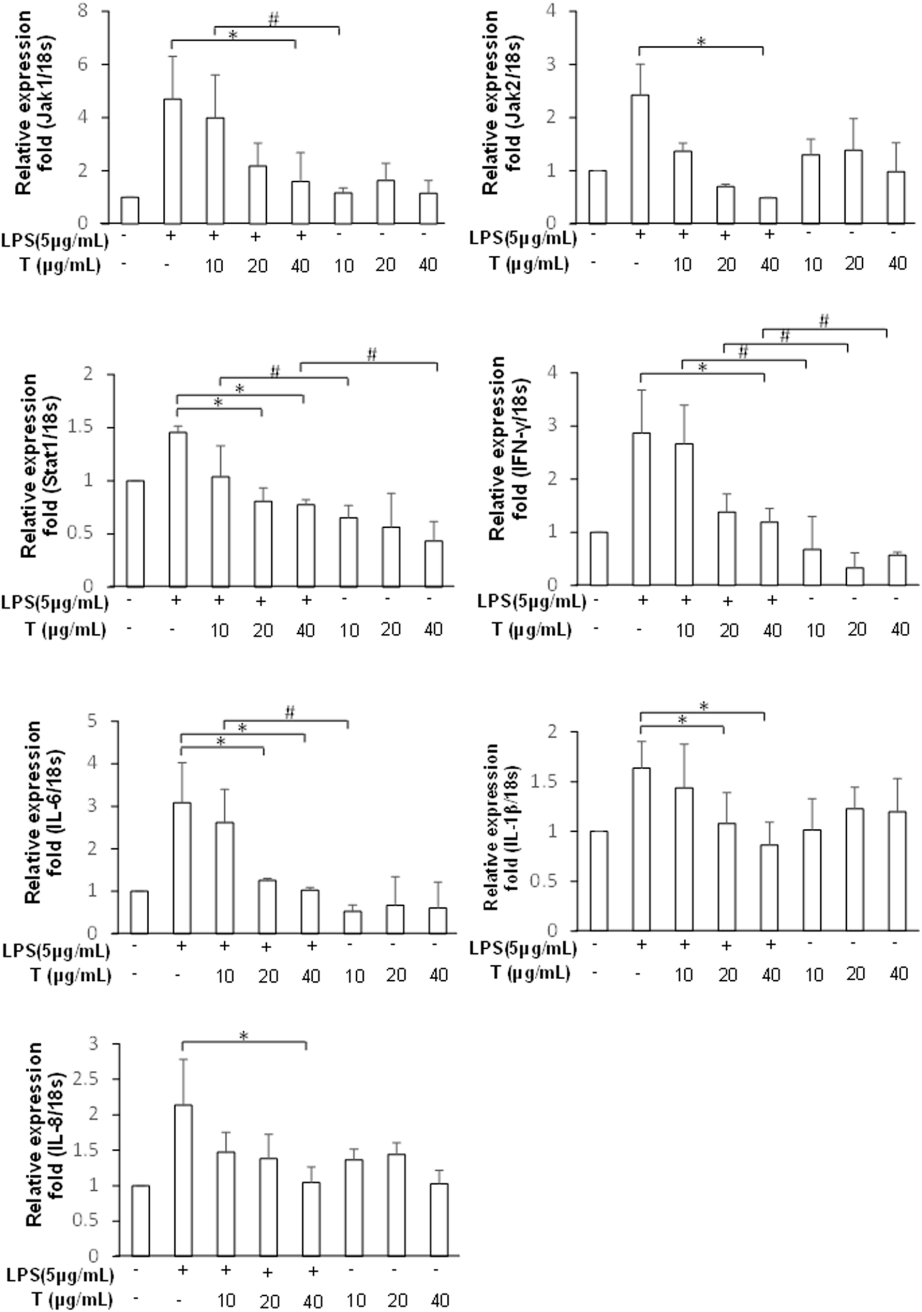
Testosterone downregulated the mRNA levels of JAK/STAT1 signaling pathway components in LPS-stimulated inflammation of prostate cells. Total RNA isolated from treated cells was analyzed using qPCR. The mRNA levels of *JAK1*, *JAK2*, *STAT1*, *IFNG*, *IL-6*, *IL1B*, and *IL-8* were detected in LPS-stimulated prostate cells treated with different doses of testosterone (T) (10, 20, and 40 μg/mL) for 24 h. Testosterone alone (10, 20, and 40 μg/mL) treatments for 24 h were performed as controls. Data from 3 separate experiments were expressed as mean ± SD when normalized to the expression of the internal control gene for the 18S ribosomal RNA. * p < 0.05, compared to the respective positive control groups; #: p < 0.05, when compared between two indicated groups. Cells stimulated with LPS alone (5 μg/mL) were as the positive control. The expression of the untreated group was used as 1 to indicate the relative fold change in expression.

**Fig 6:**
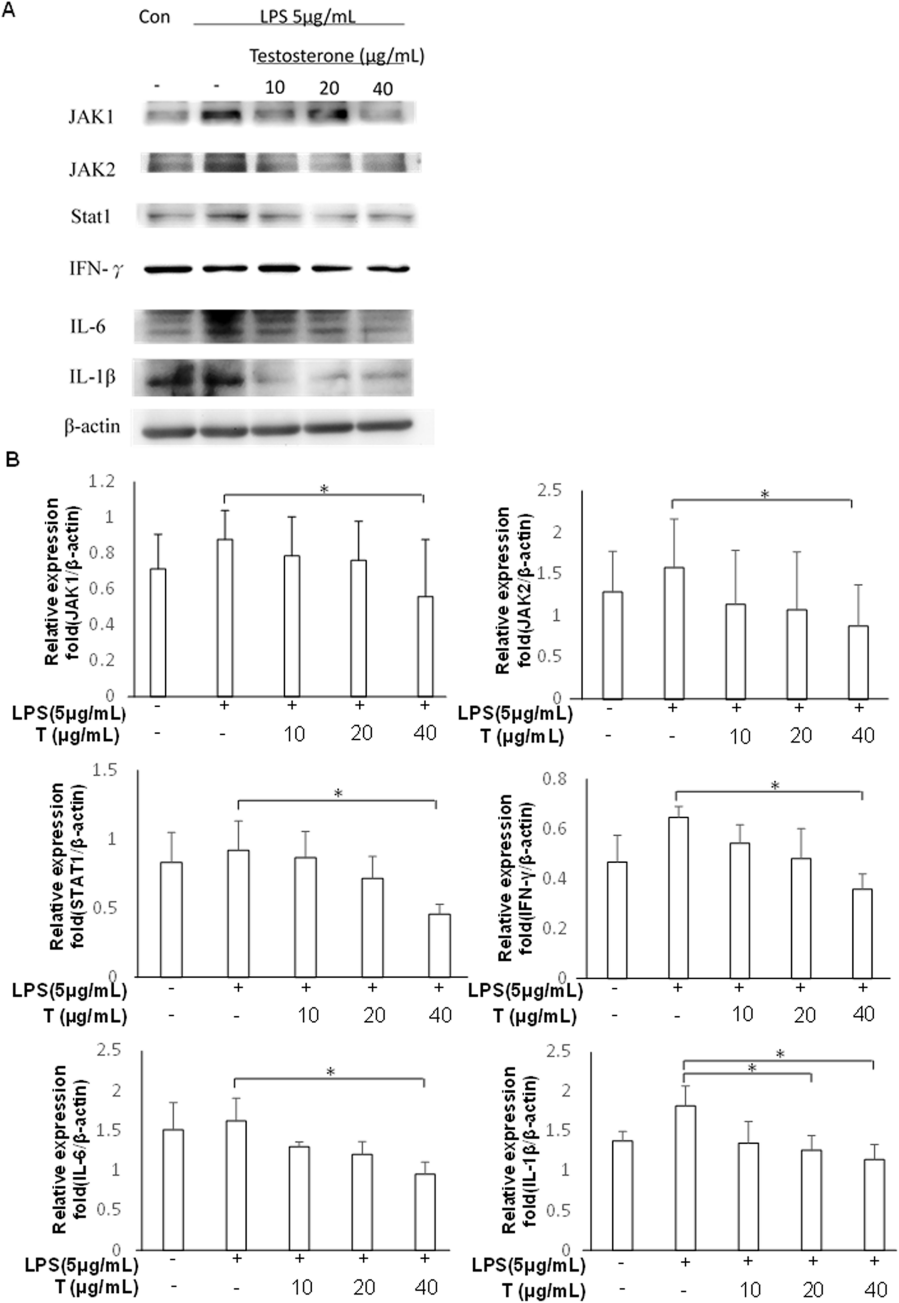
Testosterone downregulated the protein levels of JAK/STAT1 signaling pathway components in LPS-stimulated inflammation of prostate cells. PZ-HPV-7 cells were treated with different doses of testosterone (T) (10, 20, and 40 μg/mL) and stimulated with LPS (5 μg/mL) for 24 h, followed by collection of the total protein of all the treatment groups. The protein levels of JAK1, JAK2, STAT1, IFN-γ, IL-6, and IL-1β were analyzed using western blotting. All data were normalized to that of the internal reference β-actin. Results were assessed using densitometry and quantified using the ImageJ software (NIH). The results are shown as the mean ± SD from three independent experiments. * p < 0.05, compared to the respective positive control groups.

The inhibitory effects of testosterone on JAK/STAT1 signaling pathway and expression of pro-inflammatory cytokines were evaluated using LPS-stimulated PZ-HPV-7 cells. Fig 5 shows that testosterone treatment in a dose-dependent manner downregulated the expression of *JAK1*, *JAK2*, *STAT1* and *IFNG* in LPS-stimulated prostate cells (p < 0.05). Furthermore, the LPS-induced production of the mRNAs of pro-inflammatory cytokines *IL-6*, *IL1B*, and *IL-8* was significantly and dose-dependently reduced in testosterone-treated cells (p < 0.05). Testosterone also downregulated directly the INF-γ/JAK/STAT1 signaling pathway; especially, the mRNA levels of *STAT1* and *IFNG* were reduced in cells treated with testosterone alone (Fig 5, p < 0.05, compared to the positive control). However, testosterone did not suppress the production of pro-inflammatory cytokines except that of *IL-6*, the levels of which decreased slightly. Western blotting confirmed that testosterone suppressed the production of not only LPS-induced IFN-γ, JAK1/2, and STAT1, but it also substantially decreased the levels of pro-inflammatory cytokines such as IL-1β and IL-6 (Fig 6). Our data also revealed that PZ-HPV-7 cells treated with 20 μg/mL testosterone suppressed the LPS-induced JAK/STAT1 pathway and inflammatory response, and maximal effect was observed with the 40 μg/mL treatment (p < 0.05 compared to the positive controls of all the 40 μg/mL treatment groups). These results indicated that testosterone plays an anti-inflammatory role in LPS-induced prostate cell inflammation by downregulating the JAK/STAT1 signaling pathway.

### Testosterone-mediated UPEC colonization may not be related to the JAK/STAT1 pathway

**Fig 7:**
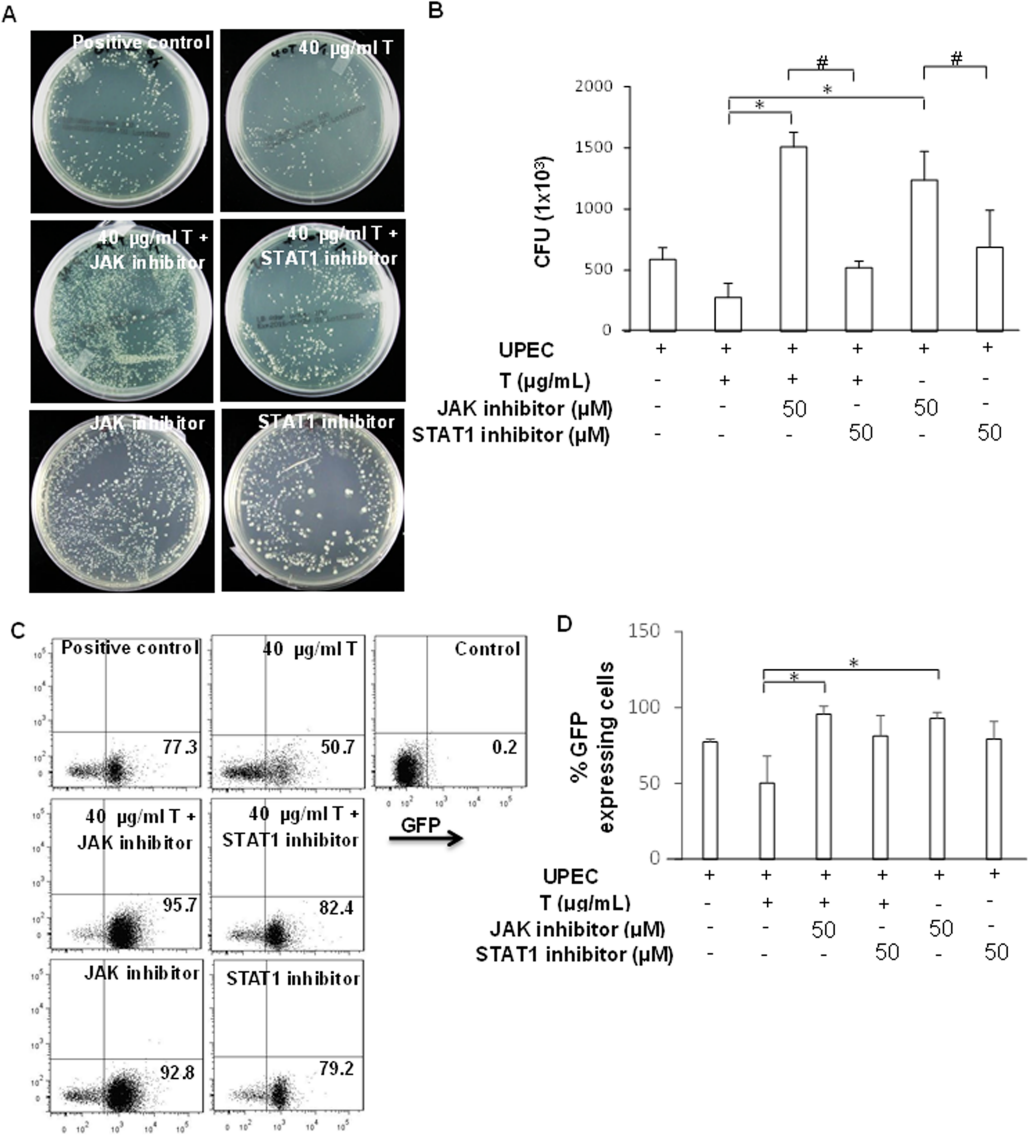
Effects of JAK and STAT1 inhibitor on testosterone-mediated suppression of UPEC colonization in PZ-HPV-7 cells. PZ-HPV-7 cells were pretreated with 40 μg/mL testosterone (T) and 50 μM JAK inhibitor or 50 μM STAT1 inhibitor for 24 h, followed by UPEC infection (MOI; 100) as described above. Cells pretreated with 50 μM JAK inhibitor or STAT1 inhibitor alone for 24 h followed by UPEC infection were used as controls. Twenty-four hours post-infection, all infected cells were lysed and plated on LB agar (A, B) or detected using flow cytometry (C) for analyzing UPEC colonization. Data are expressed as the mean ± SD from 3 separate experiments. Colony forming units (CFU) were acquired after plating ten-fold dilutions of infected cells post-lysis. Flow cytometry data are shown as dot-blots for all analyzed samples, and one representative per experiment is shown. Numbers in the right lower quadrant show the frequency of GFP-expressing cells in the quadrant of total cells. * p < 0.05, compared to the respective testosterone-treated groups. #: p < 0.05, when compared between two indicated groups.

Our data showed that testosterone exhibits an anti-inflammatory activity in LPS-induced cells by downregulating the JAK/STAT1 signaling pathway. To assess whether testosterone reduced the invasion and colonization of UPEC within prostate cells using the same pathway, we pretreated cells with testosterone and JAK or STAT1 inhibitors (50 mM/mL) to block the signaling pathway before UPEC infection as described above. Cells treated with JAK or STAT1 inhibitor alone were performed as controls. As shown in Fig 7, UPEC colonization in prostate cells co-incubated with testosterone and JAK1 or STAT1 inhibitor did not decrease 24 h after the infection. Interestingly, 24 h-pretreatment with the JAK inhibitor and testosterone rather markedly induced UPEC colonization in PZ-HPV-7 cells (p < 0.05, compared to the testosterone-treated group). Similar result was also obtained with JAK1 inhibitor alone treatment (p < 0.05, compared to the testosterone-treated group). Thus, testosterone-mediated suppression of UPEC colonization in prostate cell is not related to JAK/STAT signaling pathway, although JAK may be involved in UPEC colonization.

## Discussion

Uropathogenic Escherichia coli (UPEC) causes frequent bacterial prostatitis by refluxing the infected urine into prostatic ducts, which results in an ascending urethral infection [32, 33]. However most urinary tract infection-related studies focus on bladder cells, and studies regarding the ability of UPEC to invade, replicate, and persist in normal prostate epithelial cell are limited [3]. In this study, we established a normal prostate cell model for evaluating the ability of UPEC to invade the urothelium and colonize intercellular bacterial communities in vitro. Using this model, we demonstrated that testosterone effectively suppresses the invasion and survival of UPEC in prostate cells. In addition, testosterone-mediated inhibition of LPS-induced inflammatory responses through the JAK/STAT1 pathway has also been indicated.

A recent study indicated that UPEC is able to effectively adhere, invade, survive, and induce inflammation in an infection model of human prostate epithelial cells [4, 32]. Here we used a similar prostate cell model to evaluate whether testosterone is able to reduce the number of persistent UPEC within prostate cells 24 h post-infection. Most invading UPEC that adhere to the epithelium are cleaned by soluble cell-derived mediators such as antimicrobial peptides and chemokines [7, 34]. Our results clearly indicated that testosterone can suppress UPEC invasion and persistent survival within prostate cells in a dose-dependent manner. Although excess testosterone (above 60 μg/ mL) affected the cellular physiology and caused a significant decline in the number of prostate cells, the effective dose of testosterone required to treat UPEC infection in prostate cells is ≤ 40 μg/mL. Previous reports revealed that UPEC infection enhanced TLR4 expression and affected the cytoskeleton biology such as actin recruitment and microtubule polymerization in prostate cells, which are critical for bacterial uptake and intracellular persistence [4, 32]. TLR4 is also actively involved in bacterial invasion, and TLR4/cAMP-mediated immune function can expel UPEC from infected uroepithelial cells [35]. Leimgruber et al. showed that the presence of testosterone could downregulate TLR4 activation and prevent the dedifferentiated (myofibroblast-like) phenotype of prostate smooth muscle cells stimulated by bacterial LPS [36, 37]. They also demonstrated that testosterone reduces cytokine secretion after LPS challenge, which is consistent with our results with UPEC-infected testosterone-treated cells. This suggests that androgens might modulate the status of prostate cells for maintaining a defensive cytophysiology against UPEC infection.

Androgen supplementation has been reported to reduce the expression of inflammatory markers in prostatitis and attenuate the incidence and severity of prostatitis [38, 39]. Testosterone may reduce IL-6 and TNF-α production after LPS challenge by preventing Iκβ-alpha degradation and NF-κβ nuclear translocation [36]. Although the anti-inflammatory effects of testosterone in variety of tissues with microbial infection are well known, the mechanism of androgen action on prostate inflammatory response to bacterial LPS was not very clear. A recent study showed a critical role of the JAK/STAT1 pathway in mediating the release of high-mobility group box 1 (HMGB1) from the nucleus to the cytoplasm, which is an important inflammatory mediator for LPS-related infectious injury [40]. Effective inhibition of the JAK/STAT1 signaling may represent a critical mechanism for controlling the severity of LPS-induced inflammatory disease. In the present study, we demonstrated that testosterone effectively inhibited the production and secretion of proinflammatory cytokines including IL-6, IL-1β, and IL-8 via downregulation of JAK1/2 and STAT1 levels. A significant dose-dependent relationship between testosterone and the expression of all these factors were verified using qPCR or western blotting (Figs 5 and 6). Our data showed 40 μg/mL testosterone can effectively inhibit the JAK/STAT1 pathway and attenuate the LPS-induced inflammatory response in prostate cells. *Helicobacter pylori*, a Gram-negative bacterium, can negatively regulate IFN-γ-activated JAK/STAT signaling to mediate an immune escape strategy for attenuating antimicrobial activity. This may involve interference with the IFN-γ/JAK/STAT signaling to cause INF-γ resistance and support the persistent infection of *H*. *pylori* in gastric epithelial cells [18]. Whether UPEC uses the same mechanism of immunosuppression for persistent survival and colonization in prostate epithelial cells requires further investigation.

Inhibition of the JAK/STAT1 signaling pathway can downregulate LPS-induced HMGB1 cytoplasmic accumulation, which promotes autophagy and the suppression of bacterial phagocytosis [40–42]. Autophagy normally causes bacterial degradation; however, UPEC can block acidification and survive in lysosomes [7]. Here, we also studied whether testosterone-induced inhibition of the JAK/STAT1 pathway involves reduction in the invasiveness and persistent survival of UPEC within prostate cells. Our data revealed that blocking JAK significantly enhanced the colonization of UPEC in the prostate cells compared to the testosterone alone treatment group. Especially, treatment with the JAK inhibitor without testosterone drastically increased UPEC internalization within the prostate cells. This suggests that invasion and survival of UPEC in prostate cells might require JAK/STAT1 signaling, whereas the testosterone-mediated suppression of UPEC invasion and survival in prostate cells does not appear to utilize this pathway. A previous study showed that the level of the male-specific protein CYP2C11 is regulated by different hormones via the JAK/STAT pathway in a tissue-specific manner, with growth hormone-mediated regulation in the liver and testosterone-mediated regulation in the kidneys [43]. Subsequent experiments using a *STAT1*-overexpressing prostate cell line are required to further verify the role of JAK/STAT pathway in the UPEC invasion and survival.

In conclusion, our findings have demonstrated the suppressive effects of testosterone on the invasion and survival of UPEC and UPEC-induced inflammation in prostate epithelial cells. These findings suggest that the mechanism of action of testosterone as an anti-inflammatory mediator in prostate cells is regulated via the JAK/STAT1 signaling pathway, which may be beneficial in treating prostate inflammation. Altogether, this study has shown that testosterone can be used in the prevention and clinical treatment of prostatitis.

## Supporting information

S1 Fig The original data1 of β-actin by western blotting. (TIF)

S2 Fig The original data2 of β-actin by western blotting. (TIF)

S3 Fig The original data1 of interferon-γ by western blotting. (TIF)

S4 Fig The original data2 of interferon-γ and the original data of STAT1 by western blotting. (TIF)

S5 Fig The original data of interleukin-6 and interleukin-1β by western blotting. (TIF)

S6 Fig The original data of JAK1 and JAK2 by western blotting. (TIF)

S7 Fig Comparison of GFP-transforming UPEC with wild-type UPEC. (PDF)
