# Correction: Testosterone suppresses uropathogenic *Escherichia coli* invasion and colonization within prostate cells and inhibits inflammatory responses through JAK/STAT-1 signaling pathway

**DOI:** 10.1371/journal.pone.0190317

**Published:** 2017-12-20

**Authors:** Chen-Hsun Ho, Chia-Kwung Fan, Hong-Jeng Yu, Chia-Chang Wu, Kuan-Chou Chen, Shih-Ping Liu, Po-Ching Cheng

There are errors in the author affiliations. The affiliations should appear as shown here:

Chen-Hsun Ho^1,2^, Chia-Kwung Fan^3,4^, Hong-Jeng Yu^5^, Chia-Chang Wu^1,2^, Kuan-Chou Chen^1,2^, Shih-Ping Liu^5^, Po-Ching Cheng^3,4^

**1** Department of Urology, School of Medicine, College of Medicine, Taipei Medical University, Taipei, Taiwan, **2** Department of Urology, Taipei Medical University-Shuang Ho Hospital, Taipei, Taiwan, **3** Department of Molecular Parasitology and Tropical Diseases, School of Medicine, College of Medicine, Taipei Medical University, Taipei, Taiwan, **4** Center for International Tropical Medicine, School of Medicine, College of Medicine, Taipei Medical University, Taipei, Taiwan, **5** Department of Urology, National Taiwan University Hospital and College of Medicine, Taipei, Taiwan
