# Supplementary figures and images for: Testosterone suppresses uropathogenic Escherichia coli invasion and colonization within prostate cells and inhibits inflammatory responses through JAK/STAT-1 signaling pathway
Source: PLoS One. 2017 Jun 30;12(6):e0180244. doi: 10.1371/journal.pone.0180244 (PMC5493373; doi:10.1371/journal.pone.0180244)

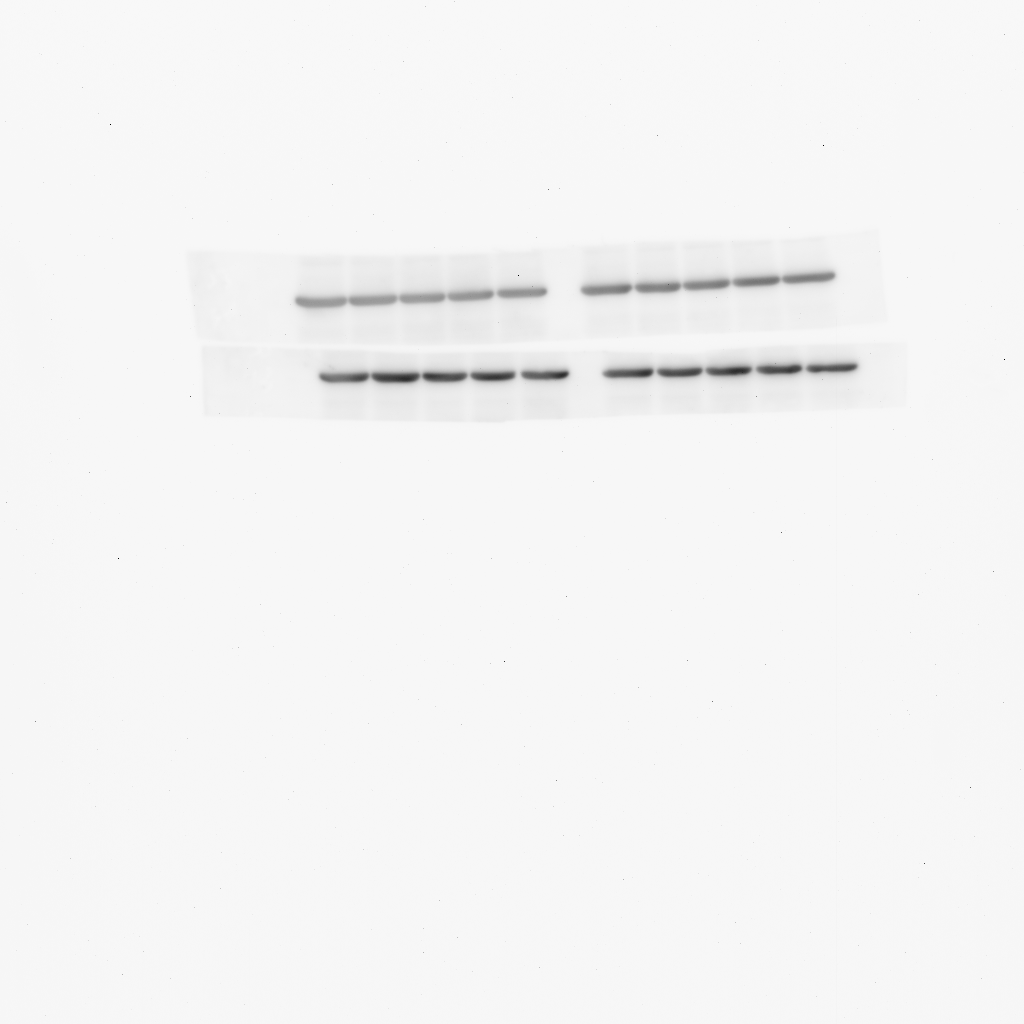

Supplement: S1 Fig — (TIF) [file pone.0180244.s001.TIF]

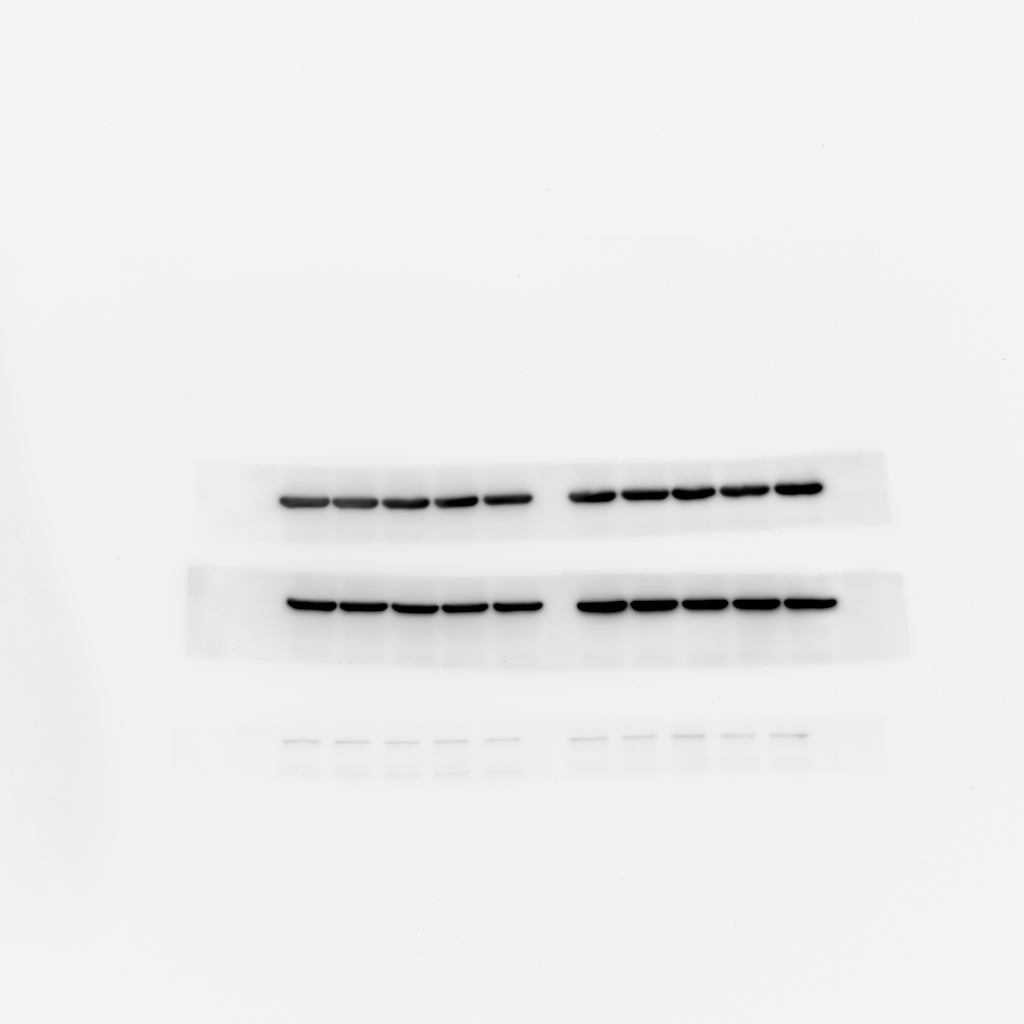

Supplement: S2 Fig — (TIF) [file pone.0180244.s002.TIF]

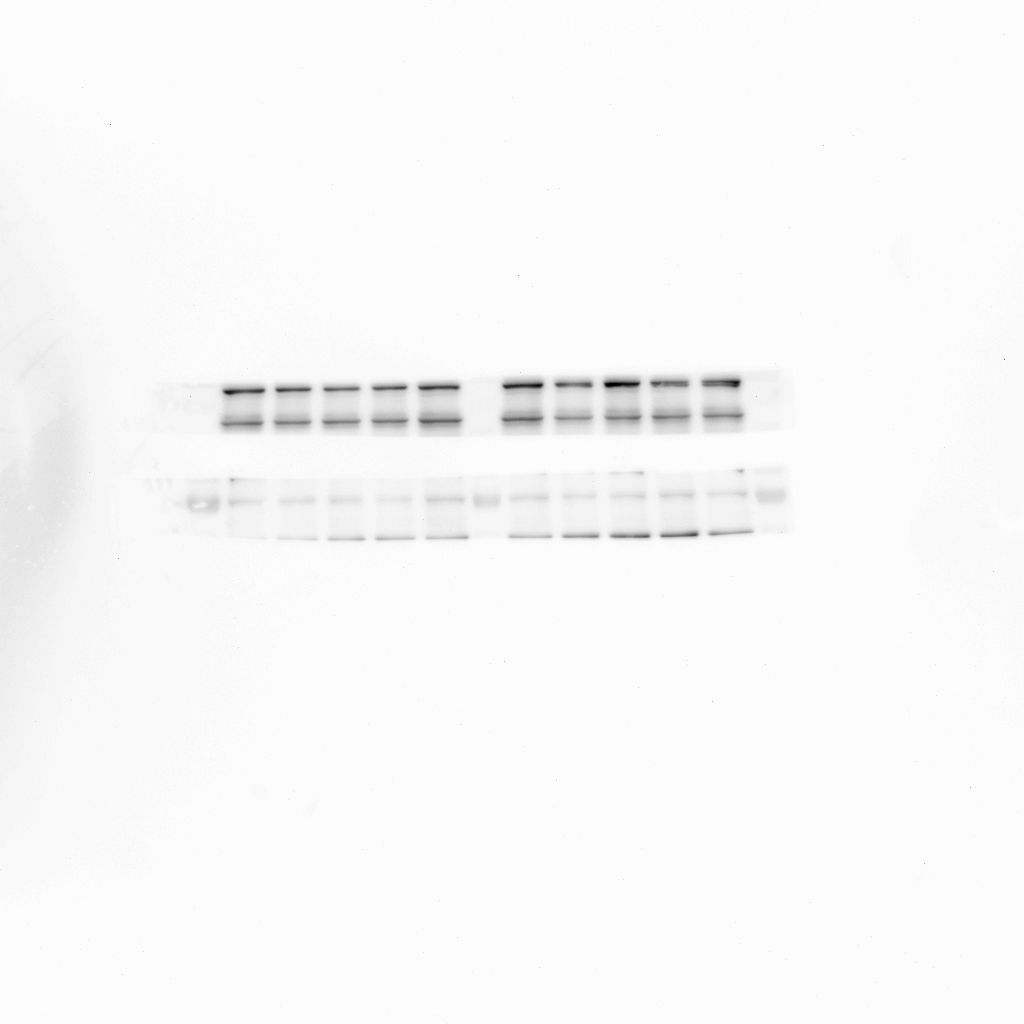

Supplement: S3 Fig — (TIF) [file pone.0180244.s003.TIF]

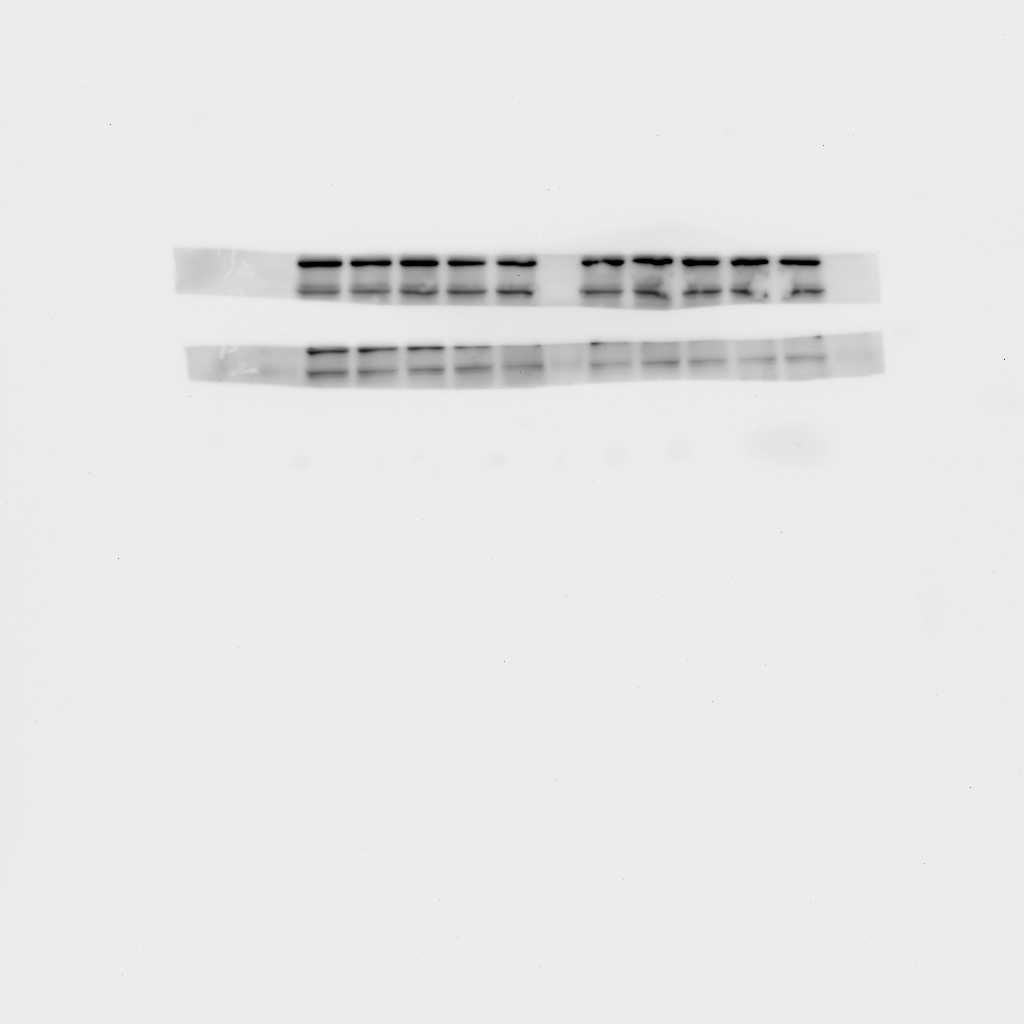

Supplement: S4 Fig — (TIF) [file pone.0180244.s004.TIF]

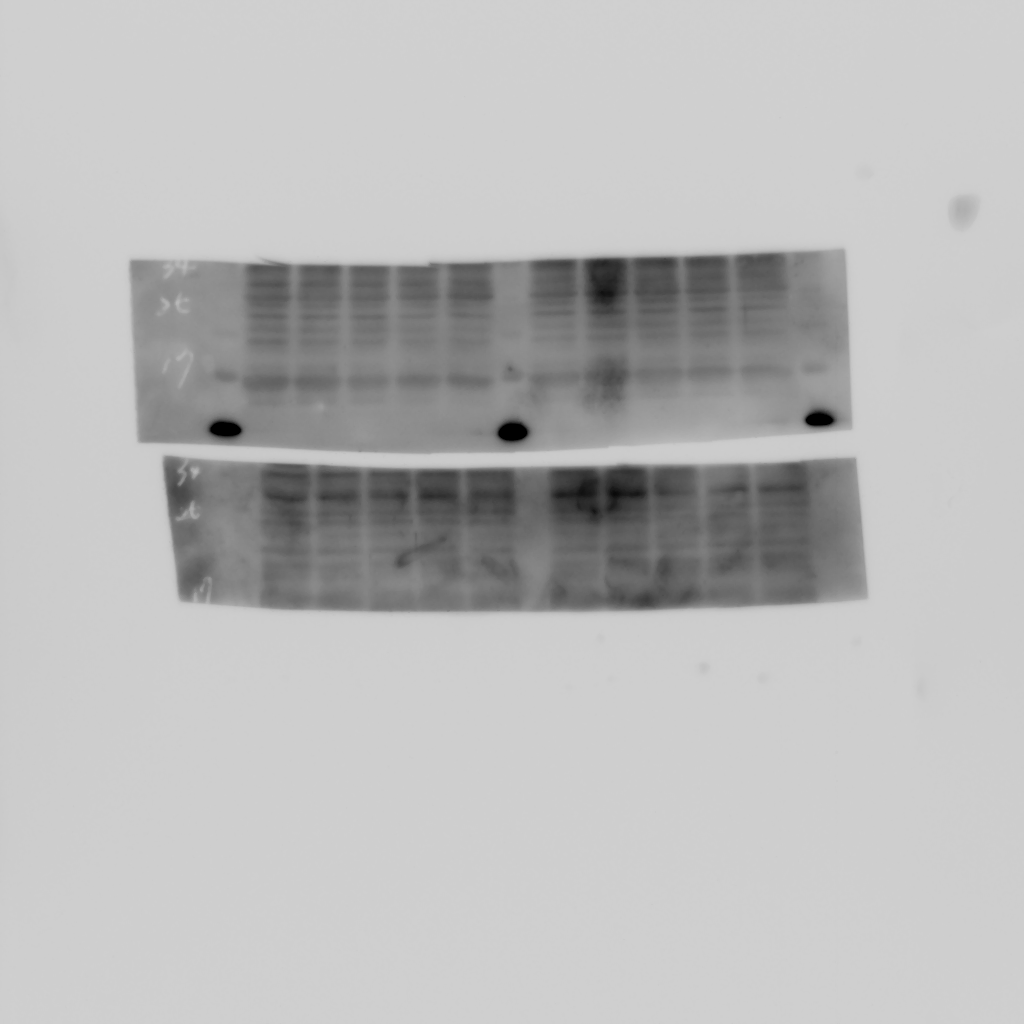

Supplement: S5 Fig — (TIF) [file pone.0180244.s005.TIF]

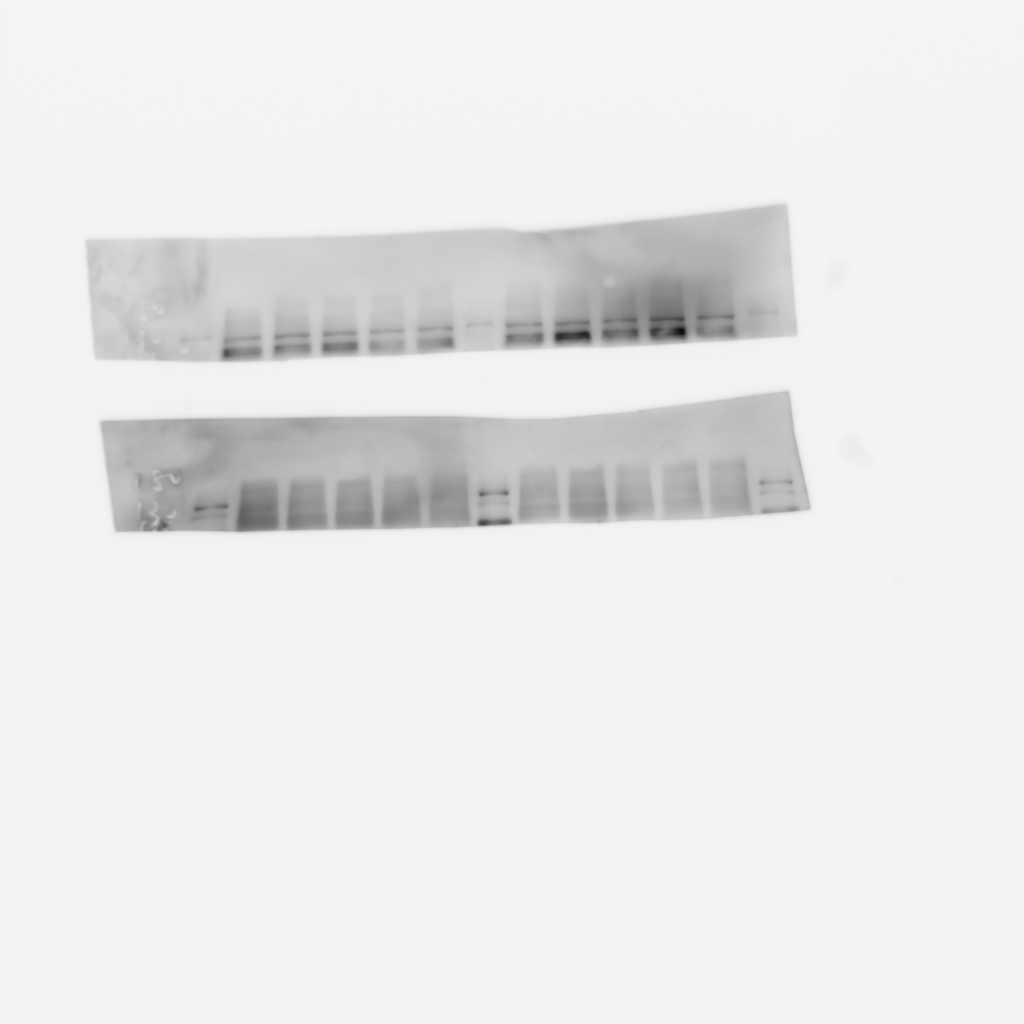

Supplement: S6 Fig — (TIF) [file pone.0180244.s006.TIF]
