# Supplementary material for: Testosterone suppresses uropathogenic Escherichia coli invasion and colonization within prostate cells and inhibits inflammatory responses through JAK/STAT-1 signaling pathway
Source: PLoS One. 2017 Jun 30;12(6):e0180244. doi: 10.1371/journal.pone.0180244 (PMC5493373; doi:10.1371/journal.pone.0180244)
Supplement: S7 Fig — (PDF) [file pone.0180244.s007.pdf]

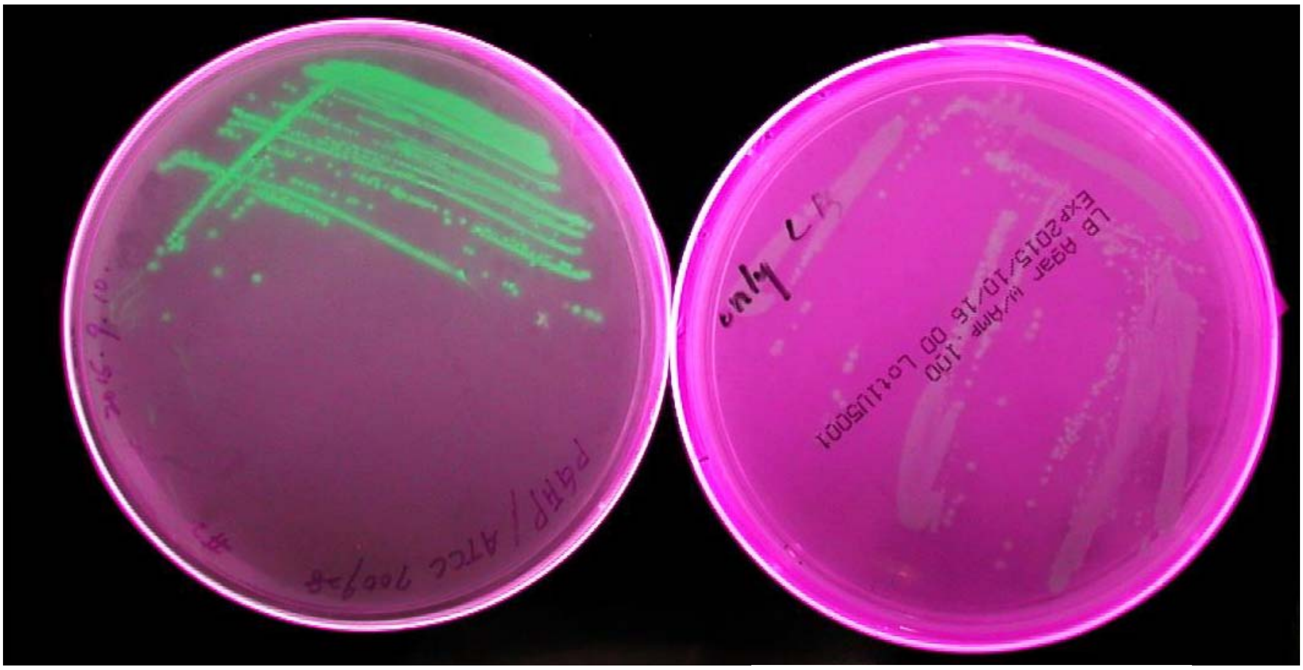

GFP transformation in UPEC

Non-transformation in UPEC

The bacterial colony can grow in both groups no matter with transformation or not, showed transformation of GFP plasmid into the UPEC does not affect the bacterial growth.
